# OMIM.org: leveraging knowledge across phenotype–gene relationships

**DOI:** 10.1093/nar/gky1151

**Published:** 2018-11-16

**Authors:** Joanna S Amberger, Carol A Bocchini, Alan F Scott, Ada Hamosh

**Affiliations:** McKusick-Nathans Institute of Genetic Medicine, Johns Hopkins University School of Medicine, Baltimore, MD 21287, USA

## Abstract

For over 50 years *Mendelian Inheritance in Man* has chronicled the collective knowledge of the field of medical genetics. It initially cataloged the known X-linked, autosomal recessive and autosomal dominant inherited disorders, but grew to be the primary repository of curated information on both genes and genetic phenotypes and the relationships between them. Each phenotype and gene is given a separate entry assigned a stable, unique identifier. The entries contain structured summaries of new and important information based on expert review of the biomedical literature. OMIM.org provides interactive access to the knowledge repository, including genomic coordinate searches of the gene map, views of genetic heterogeneity of phenotypes in Phenotypic Series, and side-by-side comparisons of clinical synopses. OMIM.org also supports computational queries via a robust API. All entries have extensive targeted links to other genomic resources and additional references. Updates to OMIM can be found on the update list or followed through the MIMmatch service. Updated user guides and tutorials are available on the website. As of September 2018, OMIM had over 24,600 entries, and the OMIM Morbid Map Scorecard had 6,259 molecularized phenotypes connected to 3,961 genes.

## INTRODUCTION

OMIM is a continuation of *Mendelian Inheritance in Man* (MIM) (1), which was published through 12 editions between 1966 and 1998. Since its inception, it has been written and curated at Johns Hopkins University, first by Victor A. McKusick and since 2002 under the direction of Ada Hamosh. From the first edition, it was maintained as a computer-based resource. This digital format enabled the National Center for Biotechnology Information (NCBI) to use it as a test bed for full-text information retrieval. In 1987, the Welch Medical Library at Johns Hopkins University School of Medicine made OMIM freely available on the internet. In 1995, OMIM was adapted for the World Wide Web by NCBI. In 2011, Johns Hopkins University sponsored the development of OMIM.org, a website that is optimized for easy retrieval and display of OMIM’s unique information even on tablets and mobile devices. A detailed description of the content scope and structure in OMIM.org is provided in the 2015 database article (2).

## OMIM ENTRIES

**Figure 1.**
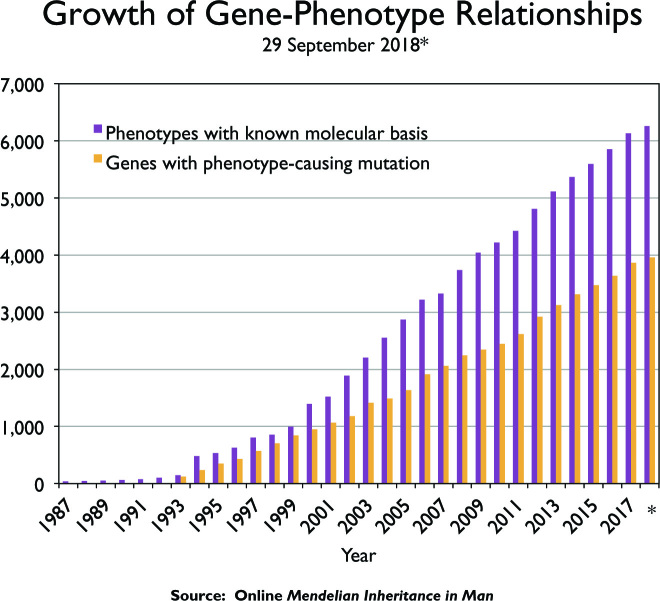
The pace of disease gene discovery as cataloged by the OMIM Morbid Map Scorecard. The current scorecard is available from https://omim.org/statistics/geneMap. As of 29 September 2018, there were over 6259 disorders spread across 3961 genes.

Throughout its history, the primary mission of OMIM has been to collect and curate knowledge on human genes and genetic disorders and traits. OMIM currently has over 24 600 entries describing over 16 000 genes and 8600 phenotypes (Table 1). All entries in OMIM are given unique and stable MIM numbers. Genes and phenotypes are described in separate entries and Allelic Variants are included in the gene entries. MIM number prefixes aid in distinguishing the content of the entry: * (asterisk) denotes a gene entry; + (plus sign) denotes an entry describing both a gene and phenotype; # (number sign) denotes a phenotype with a known molecular basis; % and *null* denote phenotypes with varying levels of supporting information for familial occurrence. Although there are a number of phenotypes in OMIM that are not ascribed to mutations in specific genes, these do not represent the totality of Mendelian disease. High-throughput and lower-cost sequencing has enabled the discovery of many previously undescribed Mendelian phenotypes. Since 2014, OMIM has added approximately 300 new phenotypes per year, and the total number of phenotypes in OMIM continues to grow. The phenotype–gene relationships are tabulated in OMIM’s Morbid Map of the Human Genome (Morbid Map). Currently, over 6200 phenotypes have been attributed to molecular alterations in over 3900 genes (Figure 1). To enable the discovery of new disease pathways and assist in organization and classification of disease, OMIM groups clinically similar phenotypes with different genetic bases into Phenotypic Series. OMIM currently has over 420 Phenotypic Series comprising over 3500 phenotypes. Several phenotypes reside in more than one series. For example, nine forms of limb-girdle muscular dystrophy are also classified as the less severe manifestation of mutations in the dystroglycanopathy genes (*FKRP, POMGNT1, POMT2*, etc.).

**Table 1.** Number of Entries in OMIM as of 29 September 2018.

MIM number prefix	Autosomal	X linked	Y linked	Mitochondrial	Totals
Gene description (*)	15 164	731	49	35	15 979
Gene and phenotypes, combined (+)	47	0	0	2	49
Phenotype description, molecular basis known (#)	4964	327	4	31	5326^†^
Phenotype description or locus, molecular basis unknown (%)	1449	124	4	0	1577
Other, mainly phenotypes with suspected mendelian basis	1656	105	3	0	1764
Totals	23 280	1287	60	68	24 695

^†^Current statistics are available at https://omim.org/statistics/entry. OMIM is updated nightly and entries that are updated can be viewed on the Update List (https://omim.org/statistics/update) and/or followed through MIMmatch. Approximately 40% of the new entries added to the database each month are new phenotypes. †Some #-sign entries represent cancer entries caused by somatic mutations in multiple genes; susceptibility to complex disease or infection (e.g., cancer, hypertension, HIV infection) with oligogenic contributions (see https://omim.org/statistics/geneMap for dissected Morbid Map counts).

The source of information for OMIM is the peer-reviewed biomedical literature. Review of high-impact journals, targeted searches of PubMed for unique phenotype–gene relationships, user-provided suggestions, and articles identified in the curation process are tracked and computationally tagged for gene, disease, and mutation concepts using PubTator (3). Articles for review and triage are pulled from the publishers’ websites to ensure timely access to the final, edited version of articles and supplemental information. Priority for inclusion into OMIM is given to papers that define new gene-phenotype relationships, significantly expand our understanding of human biology, or substantially contribute to the complete clinical characterization of a disorder and disease etiology and pathogenesis. Creation and curation of OMIM entries and data is performed by fulltime expert staff at Johns Hopkins University School of Medicine in consultation as necessary with subject domain experts. OMIM does not include all articles on a topic; to facilitate access to additional articles on a topic that are not in OMIM, a ‘reference plus’ icon appears after each paragraph. Clicking on this icon will bring up other articles in PubMed with content similar to the references in the OMIM entry paragraph.

## CLINICAL SYNOPSES

**Figure 2.**
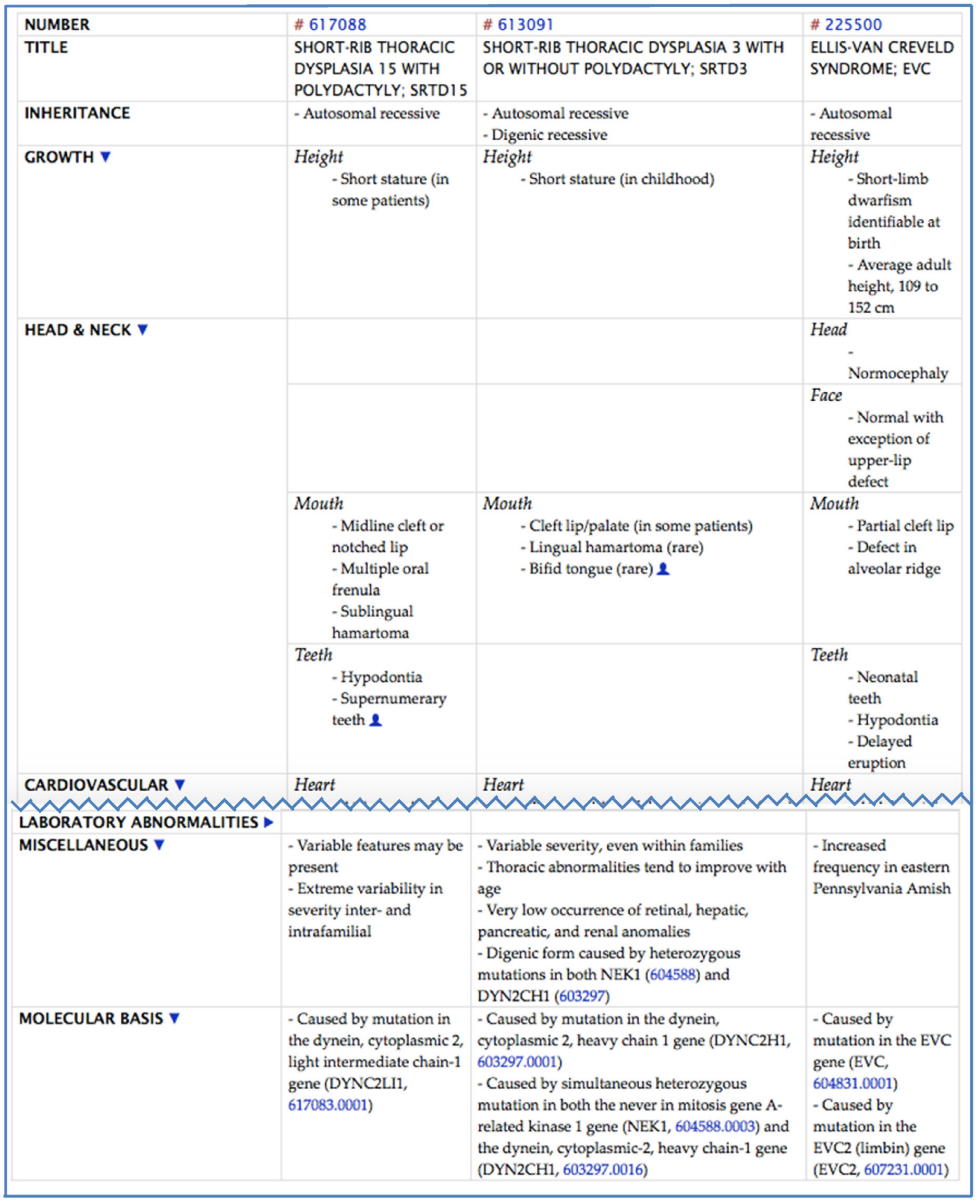
OMIM clinical synopses can be selected and viewed side-by-side to enable quick review of the similarities and differences of clinical features between and among synopses. Anatomical headings can be toggled open or closed to aid in feature comparison.

OMIM phenotype entries are linked to Clinical Synopses. These tabular lists of clinical features of a disorder are organized anatomically and are created for use primarily by clinicians. Because OMIM defines a phenotype down to the gene that is mutated in the disorder, the features in the synopsis are restricted to patients with the specific phenotype. Clinicians and researchers interested in comparing the clinical features of multiple phenotypes can now view synopses side by side (Figure 2). They can also view the Clinical Synopses of members of a Phenotypic Series in this way. These side-by-side anatomical comparisons of clinical features based on the molecular basis of the disorders inform clinicians about particular prognoses or treatments for their patients, adding to more personalized care. The clinical features in the synopses are linked to controlled vocabularies such as the Unified Medical Language System (UMLS) (4), Human Phenotype Ontology (HPO) (5), International Classification of Diseases (ICD), Disease Ontology (DO) (6) and Orphanet (7) codes. A toggle button to view these ‘Feature IDs’ is provided at the top of the Clinical Synopsis full-page view. Comparing clinical synopses is covered in OMIM’s online video tutorials and a chapter in Current Protocols in Bioinformatics (8).

## CLASSIFICATION AND NAMING OF GENETIC PHENOTYPES AND DISORDERS

Phenotypes in OMIM include single gene Mendelian disorders (e.g., Marfan syndrome, achondroplasia, Huntington disease), traits (e.g. hair and eye color), susceptibility to drug reaction (e.g., malignant hyperthermia, warfarin sensitivity), altered susceptibility or reaction to infection (herpes simplex encephalitis, progression of HIV infection to AIDS), and germline susceptibility to cancer (e.g. *BRCA1* and breast/ovarian cancer). Some recurrent deletion/duplication syndromes behave in a Mendelian fashion (e.g. Smith-Magenis and Potocki-Lupski syndromes), and OMIM has been expanding this category of entries. Some disorders that were thought to be caused by germline changes in DNA have turned out to present in the somatic mosaic state (e.g. McCune-Albirght syndrome or Proteus syndrome); these phenotypes for which a germline change would be lethal provide valuable insight into human gene function and disease pathophysiology. In this vein of understanding human disease processes, OMIM includes selected somatic mutations in cancers (e.g. the p.V660E mutation in *BRAF*, MIM:164757.0001) that are fundamental to our understanding of biology and medicine.

OMIM evaluates reports of new phenotypes in the context of those present in the catalog. Reports describing a purportedly novel phenotype are evaluated to answer the following questions: How many patients have been described in how many reports? What shared features actually define the phenotype, and how thorough are the clinical descriptions? Does this constellation of features represent a new entity? Do the different features of a disorder constitute clinical variability of a single disorder or define separate disorders? Have the same or similar features been described under a different name? Is the phenotype similar to others in OMIM? Can the phenotype be classified with any other disorders? Answering these questions must also take into account the views and possible disagreements in the genetics community as well as published nosologies. Finally, because the definition of a phenotype (constellation of features) as a genetic entity is an evolving process, the naming of a disorder in OMIM may change over time, but a MIM number remains a stable identifier. OMIM splits phenotypes by their molecular basis. Individual members of a genetically heterogeneous phenotype are grouped into Phenotypic Series. Discussion of genetic heterogeneity and the overarching phenotype description is found in the lowest numbered member of the series, and each series has a unique PS number. Clinical synopses are specific to the patients sharing the same altered gene, and their clinical synopses can be viewed side-by-side in the Phenotypic Series.

Phenotype names should be unique, enhance clinical care and classification, and be easy to communicate. Acronyms and eponyms can both serve in this capacity. When a phenotype is determined to be new to OMIM, it is assigned a number and name. If the same or similar phenotype exists in OMIM but the phenotype is caused by mutation in a different gene, the existing name is retained, followed by a serial number, and the phenotype is added to the Phenotypic Series. If the phenotype is not similar to one in OMIM, three to five of the most clinically significant features are used to create an acronym or initialism that is both informative and memorable; or, if the features are too numerous or variable, an eponym is chosen, using the names of the authors who first described or established the phenotype–gene connection, thereby ligating the eponymous phenotype to the paper(s) that described it.

**Figure 3.**
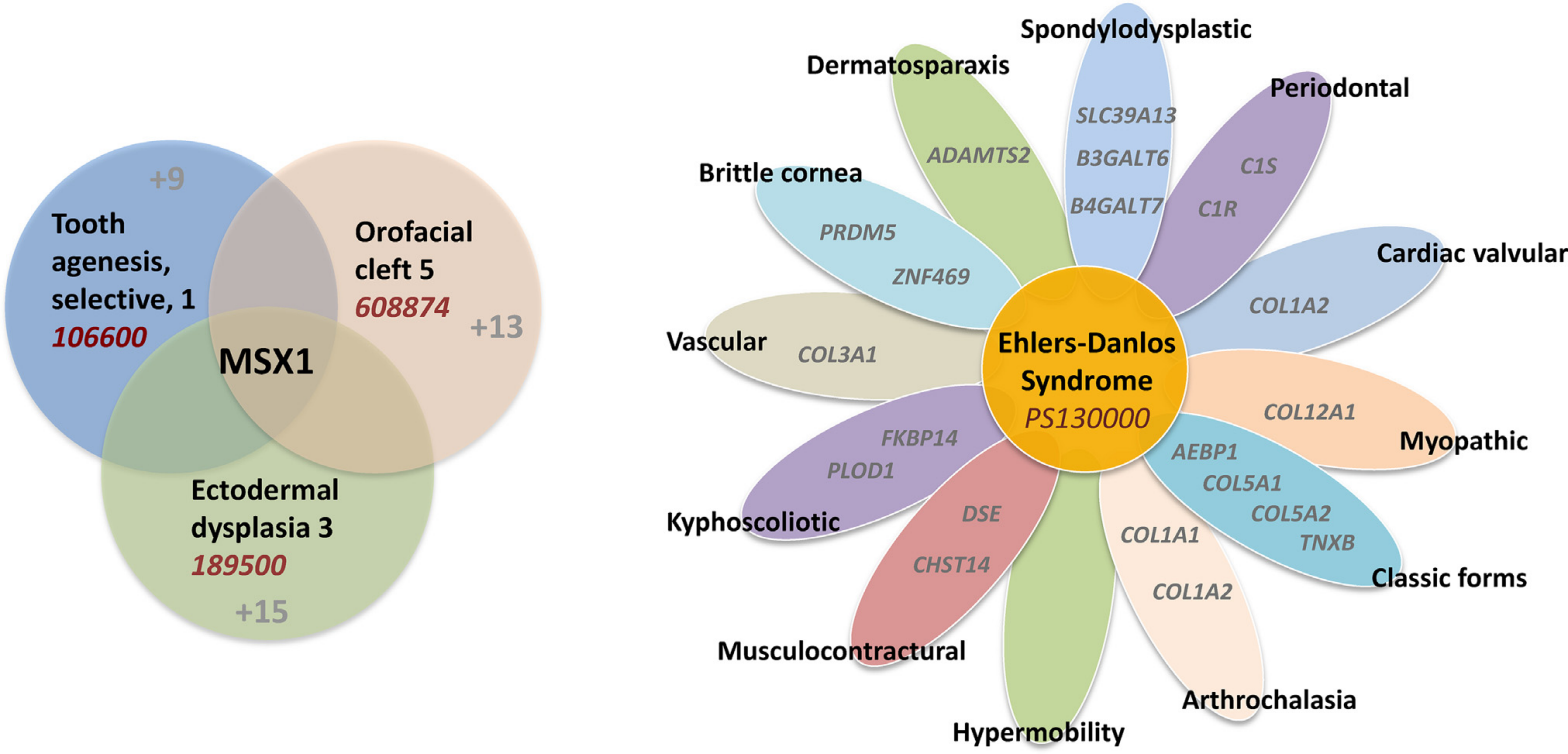
Leveraging clinical naming across molecular biology. OMIM’s approach to naming enhances analysis of the relationship between phenotypes and their molecular basis and provides new avenues of research through increased molecular understanding. Grouping of similar phenotypes into a Phenotypic Series facilitates the collection of genes underlying related clinical conditions. (**A**) Variation in the MSX1 gene can cause 3 clinically distinct disorders, each of which is part of separate Phenotypic Series. Each series lists various forms of the same phenotype caused by distinct genes (numbers in gray). (**B**) Ehlers-Danlos syndrome has been classified into 13 different clinical subtypes. The mutated gene(s) in one subtype, hypermobile, has not been found. Some of the subtypes have been found to be genetically heterogeneous, with a total of 21 different EDS syndromes. The grouping of the subtypes with their causative genes provides a unique focus for biological research.

Because phenotypes and genes are distinct concepts, their names should evolve appropriately and independently. OMIM does not name phenotypes after the gene symbol for many reasons, perhaps the most important being that patients present with clinical features, not all phenotypes have a recognized molecular basis, and many people around the world will not be sequenced. A patient has the condition whether the cause is known or not. Furthermore, more than one third of the >4000 disease genes cause more than one phenotype, each with its own unique features, prognosis, and treatment. For example, the MSX1 gene causes three different types of disorders (Figure 3A). Use of the same name for gene and phenotype confuses the literature and obfuscates text mining and machine learning. In addition, a gene name often changes with greater knowledge of the gene's function. Finally, organizing a phenotype into its various clinical subtypes allows recognition of patterns of causation (pathways and interacting proteins) that would not be possible if disorders were simply named after the genes that cause them. An illustration of the different types of Ehlers-Danlos syndrome (EDS) (Figure 3B) is an elegant example of this. Interestingly, varying components of complement 1 cause periodontal EDS while a zinc transporter and components of the O-linked glycosylation pathway cause the spondyloplastic form of EDS. The hypermobile type has not yet yielded to intense molecular interrogation, suggesting that it might be an oligogenic or multifactorial disorder.

## THE MORBID AND SYNOPSIS MAPS

OMIM uses genomic reference build GRCh38. The genes in OMIM are mapped to NCBI Entrez IDs and Ensembl IDs based on tables available at NCBI. Genomic coordinate searches can be performed in the Gene Map search option. OMIM’s gene map is used to display the phenotype–gene/gene–phenotype relationship tables, phenotypic series and gene map views. A field describing inheritance information for phenotypes is displayed in these views and is available in the genemap download file.

## EXTERNAL LINKS AND COLLABORATIONS

All OMIM entries have links to many other genomic resources as detailed in the 2015 database report. Only those links with a topical link for that gene or phenotype are listed in the External Links box to the right of the entry, and the link takes the user to the relevant page. A comprehensive list of all links is available from the Help link on the top of every page and takes one to the home page for that resource. In addition to the External Links box, there are links found in various places in an OMIM entry. OMIM includes links to many variation resources, including ExAC and gnomAD (9), as well as locus specific databases registered with the HGVS. All OMIM variants are cataloged in ClinVar (10) and updated nightly to that resource. The ClinVar and ExAC links are available from the Allelic Variant section of an OMIM gene entry and in the Allelic Variant table view option.

OMIM is actively involved in various international efforts to classify and reorganize disease nosologies and ontologies. In 2017, OMIM participated in the reclassification of the limb-girdle muscular dystrophies (11). OMIM is an active participant in the ClinGen (12) Lumping and Splitting Work Group. (Classically, ‘lumping and splitting’ has been used to describe genetic heterogeneity of a phenotype. ClinGen, however, uses it to describe phenotypic diversity at a locus.) In addition, OMIM is working with the international Gene Curation Collaboration (GenCC), a group of curated resources working to harmonize terminology to define the nature of different gene-disease relationships.

## DATA DOWNLOAD AND API

Research and educational use of OMIM is encouraged. A table linking OMIM genes to NCBI and Ensembl IDs (mim2gene.txt) is available without registration from the Downloads link on every OMIM.org web page. The gene map file containing gene–phenotype relationships including inheritance of the phenotype and a file of MIM entry titles and numbers are available from a secure HTTPS server after registration. Single-user academic, non-profit, and governmental agencies can obtain access after registering with an institutional email address. For-profit companies or anyone planning to redisplay or incorporate OMIM data into software must secure a license to ensure attribution and integrity of data use.

## OUTREACH AND TUTORIALS

The MIMmatch service available at OMIM.org provides an easy way for users to follow updates to genes and phenotypes of interest, stay current on new disease-gene relationships, find other scientists with similar interests, and save searches. In addition, MIMmatch users are permitted to download up to 1000 entries. Currently MIMmatch has over 2200 registrants. The names of MIMmatch users are not shared with any third party, and no more than one e-mail notification per day is sent. Users need to register with a valid e-mail address and confirm the registration to activate the account. OMIM’s help menus provide answers to Frequently Asked Questions, detailed search and API help. These documents also give an overview of the data structure of OMIM. A detailed guide to searching OMIM is now provided in a chapter in Current Protocols (8), and video tutorials on searching OMIM and using MIMmatch are available from multiple links on the OMIM.org website.

For over 50 years, OMIM has been a foundational resource for clinicians and researchers in molecular biology, genetics and genomics and medicine. With over two million worldwide users per year and over one million users returning over 100 times a year, OMIM remains current and authoritative through its careful selection, review and curation of the scientific literature. OMIM is the primary source of information on the evolving knowledge of the relationship between genes and disease. The free-text, structured format provides the flexibility necessary to explain the nuances of these relationships as well as to describe newly identified biological and pathological processes underlying them. As genomics becomes more integral to all of medicine, the unparalleled breadth and richness of description of human phenotypes and genes in OMIM will provide expert and timely support to clinicians and researchers in diverse scientific fields.
